# Validation of Embedded Experience Sampling (EES) for Measuring Non-cognitive Facets of Problem-Solving Competence in Scenario-Based Assessments

**DOI:** 10.3389/fpsyg.2019.01200

**Published:** 2019-05-24

**Authors:** Andreas Rausch, Kristina Kögler, Jürgen Seifried

**Affiliations:** ^1^Economic and Business Education – Workplace Learning, Business School, University of Mannheim, Mannheim, Germany; ^2^Business Education, University of Hohenheim, Stuttgart, Germany; ^3^Economic and Business Education – Professional Teaching and Learning, Business School, University of Mannheim, Mannheim, Germany

**Keywords:** embedded experience sampling, competence assessment, non-cognitive facets, problem solving, computer-based assessment, scenario-based assessment, business simulation

## Abstract

To measure non-cognitive facets of competence, we developed and tested a new method that we refer to as Embedded Experience Sampling (EES). Domain-specific problem-solving competence is a multi-faceted construct that is not limited to cognitive facets such as domain knowledge or problem-solving strategies but also comprises non-cognitive facets in the sense of domain-specific emotional and motivational dispositions such as, for instance, interest and self-concept. However, in empirical studies non-cognitive facets are usually either neglected or measured by generalized self-report questionnaires that are detached from the performance assessment. To enable an integrated measurement, we developed the EES method to collect data on non-cognitive facets during scenario-based low-stakes assessments. Test-takers are requested to stop at certain times and spontaneously answer short items (EES items) regarding their actual experience of the problem situation. These EES items are embedded in an EES event that resembles typical social interactions with non-player characters. To evaluate the feasibility and validity of the method, we implemented EES in a series of three studies in the context of commercial vocational education and training (VET): A feasibility study with 77 trainees, a pilot study with 20 trainees, and the main study with 780 trainees who worked on three complex problem scenarios in a computer-based office simulation. In the present paper, we investigate how test-takers perceived the EES events, and whether social desirability biased their answers, and investigate the internal structure of the data and the relationship between EES data and data from several other sources. Interview data and survey data indicated no biases due to social desirability and no additional burden for the test-takers due to the EES events. A correlation analysis following the multitrait-multimethod approach as well as the calibration of a multidimensional model based on Item Response Theory (IRT) also supported the construct validity. Furthermore, EES data shows substantial correlations with test motivation but almost zero correlations with data from generalized retrospective self-report questionnaires on non-cognitive facets. Altogether, EES offers an alternative approach to measuring non-cognitive facets of competence under certain conditions. For instance, EES is also based on self-reporting and thus might not be suitable for high-stakes testing.

## Introduction

Problem-solving competence has gained increasing attention in educational science as well as in vocational education and training (VET) and professional development. In vocational and professional contexts, problem-solving competence is important because of a general trend toward higher-order skills owing to the ongoing automatization and outsourcing of routine tasks that not only affect blue-collar work in production lines but also white-collar work (e.g., Brynjolfsson and McAfee, 2014; Frey and Osborne, 2017). Problem solving is considered to be an orchestration of cognitive, metacognitive, and non-cognitive processes in order to find an initially unknown way of bridging the gap between an actual state and a desired state (Dörner and Funke, 2017). Hence, unlike routine action, problem solving is by definition strenuous and problems usually evoke negative emotions that have to be dealt with. Altogether, problem solving is enhanced by motivation, excitement, perseverance, frustration tolerance, emotion regulation, (mild) positive affect, self-confidence, and so forth (Sembill, 1992; Frensch and Funke, 1995; Sugrue, 1995; Isen, 2008; Hannula, 2015; Schoppek and Fischer, 2015). Consequently, problem-solving competence also comprises non-cognitive dispositions which are also seen to be part of competence in general and work competence more specifically (Weinert, 2001; Rychen and Salganik, 2003; Kanfer and Ackerman, 2005). Nevertheless, the assessment of competencies is usually limited to cognitive aspects such as the reproduction or application of domain knowledge. We argue that a more holistic assessment of problem-solving competence should result in a competence profile that also comprises non-cognitive facets (Sembill et al., 2013; Rausch and Wuttke, 2016). The lack of holistic measurement approaches has led us to develop an experience sampling procedure which builds on the integration of emotional and motivational self-reports into computer-based competence assessments. It is referred to as Embedded Experience Sampling (EES) and has been created to capture the non-cognitive dimension of problem solving *in situ*. This contribution outlines the characteristics and implementation of EES and presents findings concerning its validity gained by conducting three empirical studies throughout the developmental process.

### Non-cognitive Facets of Problem-Solving Competence

In his seminal report, Weinert (2001) developed a broad definition of action competence as a combination of “intellectual abilities, content-specific knowledge, cognitive skills, domain-specific strategies, routines and subroutines, motivational tendencies, volitional control systems, personal value orientations, and social behaviors” (Weinert, 2001, p. 51). He pointed out that “performance in specific situations depends on more than cognitive prerequisites” (Weinert, 1999, p. 19). Similarly, Kanfer and Ackerman (2005) consider knowledge, skills, abilities, motivation, personality, and self-concept as components of work competence. Furthermore, within research on problem solving, there is a broad consensus that besides the significance of domain-specific knowledge, problem solving is also enhanced by “… some non-cognitive factors such as self-confidence, perseverance, motivation, and enjoyment” (Frensch and Funke, 1995, p. 21). Within the framework of problem solving introduced by the National Center for Research on Evaluation, Standards, and Student Testing (CRESST), problem-solving competence comprises motivation (further divided into effort and self-efficacy) along with cognitive facets (Herl et al., 1999). Similar definitions are found in research on mathematical problem solving (Verschaffel et al., 2012; Schoenfeld, 2013). There is no universally accepted definition of the term “non-cognitive” (Duckworth and Yeager, 2015) just as there is no such definition of “cognition” (Neisser, 1967). Any attempt to distinguish cognitive from non-cognitive constructs remains artificial, but facilitates the understanding and analysis of their interdependence (Weinert, 1999).

When solely focusing on the assessment of cognitive facets of competence, it is implicitly assumed that test-takers invest maximum effort to perform as well as possible. Test performance is interpreted as maximum performance in the sense of Cronbach (1960) and thus varying test motivation threatens the validity of the assessment. It is well-known that in testing for intelligence and in international large-scale studies, test motivation exerts an influence on achievement (Butler and Adams, 2007; Duckworth et al., 2011). Eklöf (2010) points out that an achievement test score is a function of “skill and will.” Correspondingly, including non-cognitive facets in the definition and modeling of competence moves the construct to be measured from “can do” to “will do” (Kanfer and Ackerman, 2005; Cortina and Luchman, 2012); or, respectively, from maximum performance to typical performance in the sense of Cronbach (1960). Consequently, emotions and motivation no longer represent construct-irrelevant variance, but are a manifest result of latent non-cognitive facets of competence which has to be considered in the measurement. Regarding convergent validity, data of non-cognitive facets of competence should be correlated with measures of test motivation.

Based on a literature review, we developed a competence model that distinguishes knowledge application, action regulation, self-concept, and interest as components of domain-specific problem-solving competence (Table 1). We further defined several facets within each of the components. These facets are arranged alongside an ideal problem-solving process and are intended to guide the measurement of problem-solving competence (Rausch and Wuttke, 2016).

**Table 1:** Model of domain-specific problem-solving competence (Rausch and Wuttke, 2016, p. 177).

Four components of competence	Thirteen facets of competence			
(A) Knowledge application(cognition)	Identifying needs for action and information gaps	Processing information	Coming to well-founded decisions	Communicating decisions appropriately
(B) Action regulation(metacognition)	Planned (well-structured) action	Persistence (focused action)		Retrospective action control
(C) Self-concept(expectancies)	Situational confidence in one’s competence	Ambiguity/uncertainty tolerance		Situational confidence in one’s solution
(D) Interests(valences)	Personal interest in the problem context/content	Maintaining positive and active emotional states		Interest in the progress of/in learning from the problem


The non-cognitive components (self-concept and interest) mirror the expectancy-value theory of achievement motivation (Wigfield and Eccles, 2000) and the control and value appraisals of achievement motivation (Pekrun, 2006), respectively. Confidence in one’s own competence when confronted with a domain-specific problem, tolerating ambiguity and uncertainty, and having confidence in one’s own solutions concerning domain-specific problems are defined as facets of a domain-specific self-concept. Being interested in the context of a domain-specific problem, maintaining positive and active emotional states while working on a domain-specific problem, and being interested in the progress of and learning from these problems are defined as facets of domain-specific interest.

### Modeling and Measuring Non-cognitive Facets of Competence

Based on a multidimensional understanding of competence, a crucial question is how non-cognitive facets are measured. Two basic options in dealing with the multidimensionality of the construct can be distinguished (Sembill et al., 2013).

#### Multifaceted Competence Model With Fragmented Measurement

Following this very common approach, non-cognitive facets are part of a multifaceted construct of competence but are measured separately, usually by administering retrospective self-report questionnaires. Those self-reports remain detached from the actual performance. In general, self-reports are considered face-valid (Debus, 2000) but there is plenty of research that stresses several threats and biases regarding the validity of decontextualized retrospective self-reports on emotion and motivation (van Reekum and Scherer, 1997; Robinson and Clore, 2002; Novak and Johnson, 2012; Schwarz, 2012). Furthermore, in their investigation of the empirical relation between intelligence and problem solving, Wittmann and Süß (1999) point to the “Brunswik asymmetry” named after Brunswik (1956) in order to explain the poor prediction of problem solving via intelligence. This poor relation is due to an asymmetry in the content and breadth of the predictor (intelligence) and the criterion (problem solving), because the former is a very broad construct, while the latter is derived from a contextualized performance task. The same argument holds true for the relation of problem solving and non-cognitive facets if non-cognitive facets are measured through general self-report questionnaires which are detached from problem solving (Rausch et al., 2016; Rausch, 2017). This approach may lead to an underestimation of the importance of non-cognitive competence facets (Dermitzaki et al., 2009; Sembill et al., 2013).

#### Multifaceted Competence Model With an Integrated Measurement

Following an integrated approach, the measurement of non-cognitive facets is integrated into the performance assessment. Regarding the differentiation of state and trait, recurrent situational emotional states are interpreted as the dispositional core of a trait emotion (Diener and Lucas, 2000). Just as the assessment of cognitive facets of competence is based on the repeated measurement of manifest performance, the suggested *in situ* assessment of non-cognitive facets is based on the repeated measurement of emotional states in the context of different problem scenarios. A multitrait-multimethod approach (MTMM; Campbell and Fiske, 1959) can be applied to investigate the internal or construct validity of such an approach. The multiple problem scenarios constitute different methods and the various non-cognitive facets (see Table 1) constitute different traits. According to MTMM (Podsakoff et al., 2003), higher correlations between the same traits across different scenarios (monotrait-heteromethod) than between different traits within one scenario (heterotrait-monomethod) indicate internal or construct validity.

### Embedded Experience Sampling to Measure Non-cognitive Facets of Competence

Our empirical approach to measuring non-cognitive facets of competence is inspired by the Experience Sampling Method (ESM) which was introduced by Csikszentmihalyi and Larson (1987, p. 526) as “an attempt to provide a valid instrument to describe variations in self-reports of mental processes.”. In ESM, participants are repeatedly requested to report their emotional states over a period of time. Different types of ESM have been established (Scollon et al., 2003, p. 7ff.): Signal-contingent sampling requires participants to complete self-reports when prompted by a randomly-timed signal (e.g., twice a day). Event-contingent sampling requires participants to complete self-reports whenever a predefined event occurs (e.g., in case of problems). Interval-contingent sampling uses constant time-intervals. The Continuous State Sampling Method (CSSM) is a special case of such time-sampling ESM with very short intervals of only 5–10 min. CSSM has been developed and applied in the context of classroom research (Sembill et al., 2008; Conrad and Schumann, 2017; Kärner et al., 2017; Kögler and Göllner, 2018). CSSM is also used for validating our own approach.

Our development of Embedded Experience Sampling (EES) builds on traditional ESM. In order to measure the non-cognitive facets in computer-based tests on problem-solving competence, EES aims at collecting self-report data on non-cognitive facets *in situ* and furthermore integrates these self-reports into the storyline of authentic problem scenarios. Test-takers are briefly interrupted during the test and requested to answer short questions (EES items) regarding their momentary experience. These EES items are embedded into the test situation in authentic EES events that resemble ordinary social interaction at the workplace (e.g., a colleague asks how one is doing). Closed-ended questions were used in order to spare the test-takers the time they would need to write down their answers. Furthermore, they improve the comparability of the answers and facilitate the implementation of EES in large-scale assessments regarding psychometric scaling. EES items focus on difficult to monitor non-cognitive competence facets such as interest, attitudes, commitment, and self-concept.

A similar approach was applied in PISA 2006 as an “embedded science interest assessment”. Directly after working on selected test items regarding science competence, the participants were requested to rate their situational interest in the prior item context. The data were calibrated in Item Response Theory (IRT) models to assess trait interest (Drechsel et al., 2011). However, few such approaches are so far known to the authors. Furthermore, the EES approach differs from the PISA approach because in PISA the items were not embedded into the “storyline” of the assessment. A further example for integrating experience sampling into a complex assessment is the “affect self-report device” applied to the game-based learning environment “Crystal Island.” During their interaction with the learning environment, test-takers received an in-game prompt asking them to report on their cognitive and emotional states. These status updates were described as part of an in-game social network (Sabourin and Lester, 2014). The “affect self-report device” is embedded in the sense of EES, but it was not designed to measure non-cognitive traits as part of a competence assessment.

Any sampling of self-reported experiences *in situ* faces limitations: for instance, social desirability may affect individuals’ responses and possibly lead to a bias in the psychometric data in terms of construct-irrelevant variance (Messick, 1994). In this context, the criteria of cognitive validity (Pellegrino et al., 2016) or construct validity (Messick, 1994), respectively, require that participants do not consciously deliberate about whether a particular answer would be more socially desirable but only answer according to their actual situational experience. Following the argument of Reis (2012), measuring non-cognitive facets within the problem-solving process promotes ecological validity, given that the problem scenarios and the EES events are representative of daily work. Furthermore, biases due to social desirability might decrease in EES compared to retrospective self-reports, due to the concurrent cognitive load and time pressure during the problem-solving process (Stodel, 2015). However, the repeated sampling of subjective states may also cause reactivity and reactance, for better or worse, because on the one hand it constitutes a disruption and on the other hand it may also trigger reflection (Csikszentmihalyi and Larson, 1987; Scollon et al., 2003; Novak and Johnson, 2012).

### Research Questions and Hypotheses

We implemented EES into test situations in three field studies and collected EES data to investigate

• How test-takers perceived the EES events (RQ1),
• Whether social desirability biased their answers (RQ2),
• The internal structure of the data (RQ3) and
• The relationship between EES data and (a) CSSM data, (b) test motivation, and (c) generalized retrospective self-reports (RQ4).

Table 2 gives an overview of the research questions and corresponding hypotheses of the field studies.

**Table 2:** Overview of the studies, the research questions, and the hypotheses.

Research questions	Study 1	Study 2	Study 3
RQ1 (ecological validity): test-takers’ perception of EES	H1a: Participants in low-stake tests do not experience EES events as an additional and unrealistic burden (interview study).	H1b: Participants in low-stake tests do not experience EES events as an additional and unrealistic burden (survey data).	
RQ2 (construct validity): social desirability	H2a: Participants in low-stake tests answer EES items without deliberating about desirable answers (interview study).	H2b: EES data and scores from the Balanced Inventory of Desirable Responding (BIDR) show zero to small correlations.	
RQ3 (construct validity): structure of the data	H3a: EES data meets the requirements of the multitrait-multimethod (MTMM) approach.		H3b: EES data meets the requirements of a multidimensional model based on Item Response Theory (IRT).
RQ4: relations between EES and			
(a) … CSSM data (convergent validity)		H4a: EES data of situational interest and CSSM data of situational interest show medium to large correlations.	
(b) … test motivation (convergent validity)		H4b: EES data of situational interest and test motivation show medium to large correlations.	
(c) … generalized retrospective self-reports (divergent validity)	H4c: EES-based scores and retrospective measures of vocational interest and self-concept show small correlations.		H4c (Replication): EES-based scores and retrospective measures of vocational interest and self-concept show small correlations.


The studies were part of the research project ‘modellng and measuring domain-specific problem-solving competence of industrial clerks’ (DomPL-IK), which was funded by the German Federal Ministry of Education and Research (Grant No. 01DB081119–01DB1123). The apprenticeship program to become an industrial clerk is the fifth most frequent of nearly 330 state-recognized apprenticeship programs in the well-respected German dual system of vocational education and training (VET). Apprenticeship programs usually require 3 years to complete and are characterized by a combination of workplace learning in the training company and classroom-based learning in state-run vocational schools. Certified industrial clerks usually work in back-office departments of industrial or service companies. A general description of the research project and selected results have been published in Rausch et al. (2016).

In the present article, we focus on the development and validation of the EES approach by analyzing EES data from two pilot studies and the main study. In a first feasibility study, we investigated how participants perceived the EES events, whether social desirability played a role, whether the EES data met the requirements of the MTMM approach, and how EES data were correlated to retrospective measures of interest and self-concept. The aims of the second pilot study was to test the computer-based office simulation that, for the first time, also included a computer-based implementation of EES events. Additional data were collected to investigate the subjective experience of the EES, social desirability in EES responses, and the relation to CSSM data and test motivation. Finally, the computer-based assessment of domain-specific problem-solving competence was implemented in a large-scale study with almost 800 participants in vocational schools in six federal German states. The resulting EES data were calibrated in a psychometric model based on Item Response Theory (IRT). Parts of this final step of the test development are published in Rausch et al. (2016). The studies within the research project have been approved by the responsible ministries of education and the responsible commissions of data protection of the respective German Federal States as well as by the Ethics Committee of the Otto-Friedrich-University of Bamberg (Otto-Friedrich-University Bamberg, Bamberg, Germany).

## Study 1: Feasibility Study of Implementing EES Events Into Authentic Problem Scenarios

### Materials and Methods

#### Participants

The feasibility of implementing EES in the assessment of domain-specific problem-solving competence was investigated in a pilot study with *N* = 77 students in vocational education and training (VET) of two vocational business schools in Germany. All participants were enrolled in a 3-year apprenticeship program to become industrial clerks and were nearing the end of their 2nd year of the apprenticeship. The sample included 28 male and 49 female participants who showed a typical age distribution (*M* = 21.8; *SD* = 1.56; min = 18; max = 26). Participation was voluntary and all participants provided written informed consent.

#### Procedure

Data were collected in computer-equipped classrooms. At the beginning of the data collection sessions the researchers introduced themselves, the project, and the agenda. First, the participants completed several self-report questionnaires including scales on vocational interest and work-related self-efficacy. Next, they worked on three authentic, computer-based business problems including the completion of several EES items (for further information see Rausch, 2017). The session ended with group discussions or individual interviews about the problem scenarios and the experience of EES.

The three computer-based problem scenarios required a cost deviation analysis (30 min), a supplier selection (40 min), and a make-or-buy decision (50 min). Each scenario started with an email from a supervisor which included a problem and a variety of documents of varying relevance, transparency, and credibility. All scenarios required participants to go through multiple processes of information seeking, processing, and interpreting. To complete a scenario, the participants had to reply to the initial email with a well-founded proposed solution. The test environment provided “open book” conditions meaning that participants could look up technical terms, formulae, legal regulations etc. in a large reference work. However, they were not allowed to consult any other sources such as the internet. The participants used Microsoft Excel^®^ to work on several spreadsheet files and Microsoft Word^®^ documents to write their email reply and make notes. The problem environment was open in the sense that there was no further structure provided during the given time frame for each problem scenario. Editable documents were analyzed for each participant to assess the cognitive facets of problem-solving competence (see Table 1). For further information on the analysis of the cognitive facets see Rausch et al. (2016), Rausch (2017), and Seifried et al. (unpublished).

#### Measures

##### Embedded experience sampling (EES)

In this feasibility study, four EES events were implemented into each of the above problem scenarios. Table 3 lists the EES events, the related competence facets, and the EES items that were used. In this first application of the method, no events and items had been designed for the competence facets C2 “ambiguity/uncertainty tolerance” and D1 “personal interest in the problem context/content”.

**Table 3:** Overview of EES events, competence facets, and EES items in Study 1.

EES event (point of time)	Competence facet (see Table 1)	EES items (translated from German and condensed)
Short email response after the reception of the task (after 3 min)	Situational confidence in one’s competence (C1)	C1_1: Sender of the task requests a first quick estimation. Answer from 1 = ‘*I do not know what to do here yet’* to 4 = ‘*I know exactly what to do here.’*
Phone call from the sender of the task (after 10 min; in scenario 3 after 20 min)	Situational confidence in one’s competence (C1)	C1_2: Sender of the tasks requests a further estimation. Answer from 1 = ‘*I am afraid that I will not be able to cope with it, but I will do my best’* to 4 = ‘*I can definitely cope with it and I will do my best.’*
Short visit by a colleague (after 20 min; in scenario 3 after 35 min)	Maintaining positive and active emotional states (D2)	Friend enters the office asks how one is doing. D2_1: from 1 = ‘*not at all’* to 4 = *‘very nervous/worried.*’ (-) D2_2: from 1 = ‘*not at all’* to 4 = *‘very motivated/interested.’* D2_3: from 1 = ‘*not at all’* to 4 = *‘very irritated/annoyed.’* (-) D2_4: from 1 = ‘*not at all’* to 4 = *‘very confident/optimistic.*’
Short request from the sender of the task after the reception of the solution (after submission or after 30 min in scenario 1, after 40 min in scenario 2 and after 50 min in scenario 3, respectively)	Situational confidence in one’s solution (C3)	C3: Sender of the task asks how confident the apprentice is about her/his solution and whether the solution has to be checked before its implementation. Answer from 1 = ‘*Unfortunately*, *I did not arrive at a solution at all’* over 2 = ‘*I am afraid you should check everything in detail because I assume I made some mistakes*’ to 5 = *‘I think I found a proper solution that you do not have to check in detail again.’*
	Interest in the progress of/in learning from the problem (D3)	Participants can add none, several or all of the following statements to his/her e-mail answer. *‘I would be very happy if you could …’* D3_1: *… inform me about the final decision that you made.’ ‘* D3_2: *… give me feedback in case of any errors I made’.* D3_3: *… explain the correct procedure to me’.* D3_4: *… assign similar cases to me in the near future’.*


*EES events and EES items were the same for all of the three problem scenarios; (-) indicate inverse items. Abbreviations behind competence facets refer to competence model in Table 1; facets D1 and C2 have not been measured yet in this first study.*

**FIGURE A1:**
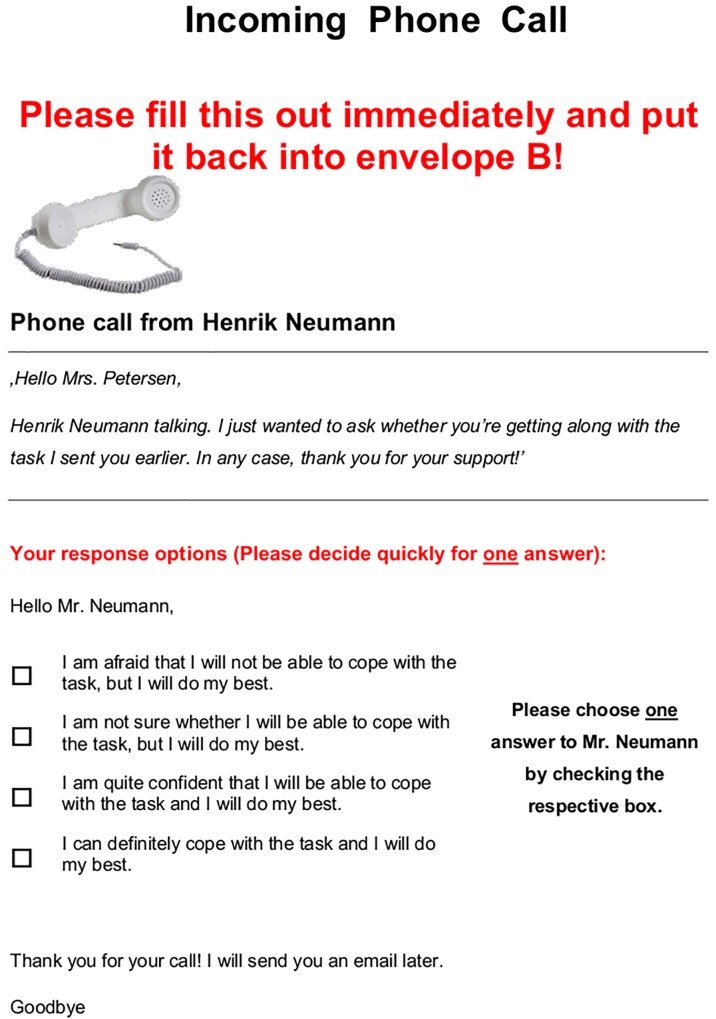
Paper-based EES event “Phone call” with one EES item (translated from German).

In this early stage of the project, EES events were paper-based and came in separate envelopes that were numbered consecutively and placed on each participant’s desk (see Appendix Figure A1 for an example). Female and male participants were provided a gender-specific version of the EES events. At predefined times during the test, participants were asked to open a particular envelope, to immediately complete the items, and to put the paper sheet back into the envelope. Altogether, 1,845 such envelopes were prepared for this study. Apparently, test efficiency was questionable in this paper-based implementation of EES.

The data of the two EES items concerning the competence facet “confidence in one’s competence” (C1) were condensed into one scale for each scenario. The internal consistencies were not satisfactory (0.57 < Cronbach’s alpha < 0.59). “Situational confidence in one’s solution” (C2) was measured with a single item (see Table 3). The data of the four EES items on the competence facet “positive and active emotional state” (D2) were condensed into one scale for each scenario. Inverse items were re-coded and a mean score was calculated for each scenario. Again, the internal consistencies were not satisfactory (0.56 < Cronbach’s alpha < 0.61). The four dichotomous EES items on the competence facet “interest in the progress of the problem” (D3) were condensed into one scale for each scenario by sum score. Thus, the scores for each non-cognitive facet ranged from 1 to 4.

##### Generalized self-reports of work-related self-efficacy and work-related interest

We administered a scale designed to measure work-related self-efficacy (Abele et al., 2000). The scale consisted of six statements that were rated on a five-point Likert scale ranging from 1 = disagree to 5 = agree (e.g., “I do not worry about work-related challenges because I can always trust my abilities.”). The internal consistency of the scale was satisfactory (Cronbach’s alpha = 0.73). An adapted and shortened version of a scale originally developed to measure dispositional interests in students (Schiefele et al., 1993) was administered. The scale consisted of six statements rated on a four-point Likert scale ranging from 1 = disagree to 4 = agree. The items assessed general interest in the current apprenticeship program (e.g., “I am sure that I have chosen an apprenticeship program which reflects my personal interests.”). The internal consistency of the scale was satisfactory (Cronbach’s alpha = 0.76).

##### Subjective experience of EES

To investigate how the participants experienced the EES, two group discussions in class (with approximately 20 participants each) and 11 individual interviews were conducted. Participants were asked how they experienced the procedure (the additional questions that came in the envelopes). They were asked whether they had deliberated about alternative responses and whether answering these questions had caused additional stress during their work on the problem situations.

#### Data Analysis

Following a multitrait-multimethod (MTMM) approach, the various facets of competence are multiple traits and the three scenarios are multiple methods. Although the variables were not normally distributed (Shapiro–Wilk tests), parametric Pearson correlations were calculated since this method is considered robust (Norman, 2010). In correlation tables, indications of significance are omitted in favor of legibility. Following Cohen (1988), correlation coefficients of 0.10 < *r* < 0.30 indicate small effects, 0.30 < *r* < 0.50 indicate medium effects, and *r* > 0.50 indicate large effects. The interview data were categorized with regard to social desirability and the additional burden of answering the EES items while working on the problem scenarios. The data was analyzed using IBM SPSS 24.

### Results

#### Descriptive Statistics

The mean values for the EES variables range between 1.71 and 3.19 on a four-step scale (see Appendix Table A1). The variable D2 (maintaining positive and active emotional states) shows high values, consistently above the value of 2.3, while variable D3 (interest in the progress of/in learning from the problem) shows much lower values. Here, the mean values only reach a value above 2.0 in scenario 2. Finally, the decrease of the mean values over time for variable C3 (situational confidence in one’s solution) is noteworthy. The mean value drops from 2.97 in scenario 2 to 1.71 in scenario 3. This finding is in line with the difficulty of the scenarios (determined by the solution rates)—scenario 2 was evaluated as the easiest one while scenario 3 showed the lowest solution rate, as expected with regard to the complexity of the scenario.

#### Test-Takers’ Perception and Social Desirability (RQ1, RQ2)

To investigate participants’ subjective experience of the EES, individual interviews and group discussions were conducted. In both group discussions the participants reacted positively to the way in which social interaction was implemented via the paper-based questionnaires and stated that such interruptions were quite realistic. Two of the 11 individually interviewed participants made similar statements when asked how they experienced these short questionnaires and added that it was an entertaining addition to the test scenarios. None of the participants reported adverse experiences. In one group discussion, a participant cautiously indicated that one could have thought about how some of the responses would appear to others. All of the 11 individually interviewed participants indicated that they answered spontaneously according to their actual experience and did not deliberate about “good answers”. Only one out of 11 participants stated that answering the EES items caused an additional burden. Altogether, the participants’ responses gave no reasons to assume biases from social desirability or any additional burden and thus they support H1a and H2a (see Table 2).

#### Multitrait-Multimethod Analyses (RQ3)

In the next step, we analyzed the structure of the data by applying a multitrait-multimethod approach. High heterotrait-monomethod correlations between different non-cognitive competence facets (traits) within a scenario (method) argue for situational influences of the scenario, while high monotrait-heteromethod correlations between the same competence facets (traits) measured in different scenarios (method) argue for trait influences. Table 4 shows the results of the MTMM analysis.

**Table 4:** Correlations between EES items within and across three problem scenarios in Study 1.

	Scenario 1				Scenario 2				Scenario 3			
	C1	C3	D2	D3	C1	C3	D2	D3	C1	C3	D2	D3
**Scenario 1**												
C1	1.00											
C3	0.32	1.00										
D2	0.34	0.43	1.00									
D3	–0.06	0.16	–0.08	1.00								
**Scenario 2**												
C1	0.29				1.00							
C3		0.24			0.40	1.00						
D2			0.20		0.42	0.40	1.00					
D3				0.71	0.15	0.07	0.10	1.00				
**Scenario 3**												
C1	0.05				0.46				1.00			
C3		0.08				0.23			0.64	1.00		
D2			0.29				0.24		0.45	0.54	1.00	
D3				0.58				0.64	0.31	0.22	0.21	1.00


*C1, situational confidence in one’s competence; C3, situational confidence in one’s solution; D2, maintaining positive and active emotional states; D3, interest in the progress of/in learning from the problem; abbreviations of the competence facets refer to the competence model in Table 1; facets D1 and C2 have not been measured yet in this first study.*

The mean correlation of all 18 heterotrait-monomethod combinations is *r* = 0.28 while the mean correlation of all 12 monotrait-heteromethod combinations is *r* = 0.33, which is consistent with the MTMM assumption. Heterotrait-monomethod correlations different from zero are plausible because the theoretical constructs are not assumed to be fully independent of each other. The monotrait-heteromethod correlations are higher which supports the assumption of internal validity and thus supports H3a (see Table 2). However, they are not much higher than the heterotrait-monomethod correlations. Internal consistency across all three scenarios and across both EES variables of self-concept was CA = 0.66 (6 variables); the respective internal consistency across all three scenarios and across both EES variables of interest was CA = 0.71 (6 variables).

#### Relations Between EES and Generalized Retrospective Self-Reports (RQ4)

Finally, by calculating mean scores across the EES variables, we received two EES-based scales, one for self-concept and one for interest. The correlations between EES-based scales and scales from generalized self-reports of work-related self-efficacy and vocational interest were close to zero and not significant (*r* = 0.05, *p* = 0.66 for self-concept; *r* = 0.04, *p* = 0.69 for interest). We hypothesized small correlations (H4c) even though the theoretical constructs are quite similar.

## Study 2: Validation Study of Responses to Computer-Based EES Events

### Materials and Methods

#### Participants

To test the computer-based implementation of EES events and the subjective experience of the EES, 21 VET students participated voluntarily in this pilot study and provided written informed consent. Eight participants were male and 13 were female; the participants were 20.3 years old on average (*SD* = 1.93; min = 18; max = 24).

#### Procedure

**FIGURE 1:**
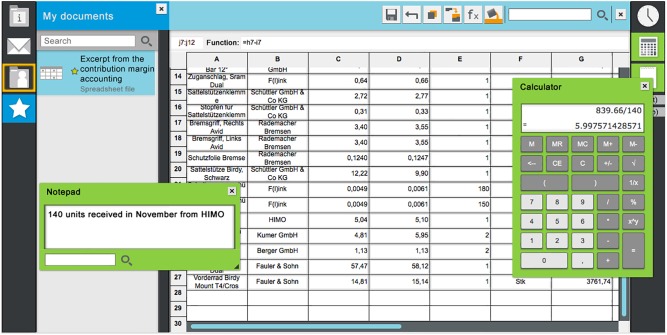
Screenshot of the office simulation (translated from German; Rausch et al., 2016, p. 8).

Data were collected in a computer-equipped classroom. At the beginning of the sessions the researchers introduced themselves, the project, and the agenda. The participants worked on one authentic, computer-based problem scenario including the completion of several EES items. In contrast to the feasibility study, the scenario in this pilot study was presented and completed in an integrated custom-built office simulation that comprised typical features of an office workplace, such as an email client, a spreadsheet application, a folder structure, a file viewer, a notepad, a calculator and so forth. Figure 1 shows a screenshot of the office simulation.

In addition to EES, data were also collected via the “Continuous State Sampling Method” (CSSM) and via a short questionnaire on test motivation and one’s experience with the EES events directly after the problem scenario. Furthermore, the participants completed a longer questionnaire that included biographic information as well as several standardized scales, one of which was applied to measure a disposition toward socially desirable responding.

#### Measures

##### Embedded experience sampling method (EES)

In this pilot study of the technological implementation, four EES events were defined. However, due to a technical malfunction the fourth EES event was not presented to the participants. Table 5 lists the remaining three EES events, the related competence facets, and the EES items that were applied.

**Table 5:** Overview of EES events, competence facets, and EES items in Study 2.

EES event (point of time)	Competence facet (see Table 1)	EES items (translated from German and condensed)
EES event 1: short email response after the reception of the task (after 3 min)	Situational confidence in one’s competence (C1)	C1_1: Sender of the task requests a first quick estimation. Answer from 1 = ‘*I do not know what to do here yet’* to 4 = ‘*I know exactly what to do here’*.
	Personal interest in the problem context/content (D1)	D1: Sender of the task asks whether tasks like this are interesting to the apprentice. Answer from 1 = ‘*Tasks like this are not interesting to me*’ to 4 = ‘*Tasks like this are very interesting to me’*
EES event 2: phone call from the sender of the task (after 10 min)	Situational confidence in one’s competence (C1)	C1_2: Sender of the tasks requests a further estimation. Answer from 1 = ‘*I am afraid that I will not be able to cope with the task, but I will do my best’* to 4 = ‘*I can definitely cope with the task and I will do my best’*.
	Ambiguity/uncertainty tolerance (C2)	C2: Sender of the task asks whether the apprentice likes to work on comprehensive tasks like this. Answer from 1 = ‘*I do not like to work on such comprehensive tasks’* to 4 = *‘I very much like to work on such comprehensive tasks*’.
EES event 3: short visit by a colleague (after 20 min)	Maintaining positive and active emotional states (D2)	Friend enters the office asks how one is doing. D2_1: from 1 = ‘*not at all’* to 4 = *‘very nervous*’. (-) D2_2: from 1 = ‘*not at all’* to 4 = *‘very curious’*. D2_3: from 1 = ‘*not at all’* to 4 = *‘very irritated*’. (-) D2_4: from 1 = ‘*not at all’* to 4 = *‘very confident*’.


**FIGURE 2:**
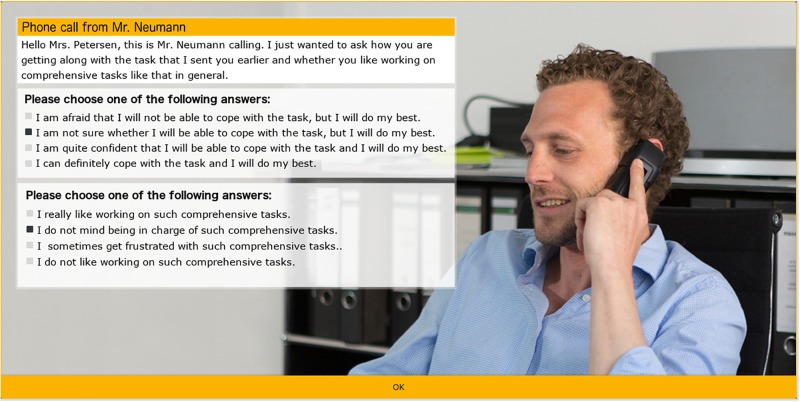
Computer-based EES event “phone call” with two EES items (translated from German; written informed consent was obtained for the publication of this image from the individual featured).

In this validation study, the EES events were also presented within the office simulation for the first time. Figure 2 shows the EES event “phone call.”

Embedded experience sampling data were condensed in the same way as in the feasibility study (Study 1), resulting in four EES variables for the competence facets shown in Table 1, C1 (confidence in one’s competence), C2 (uncertainty tolerance), D1 (interest in the problem content), and D2 (positive emotional states).

##### Continuous state sampling method (CSSM)

Continuous state sampling method data was collected during the problem scenario via mobile devices (PalmOne Tungsten^®^). In 5-min intervals, the participants were requested to rate three statements on a scale from 0 to 100. The items were: (1) *Right now, this is very interesting*. (2) *Right now, I am making great efforts*. (3) *Right now, I am making great progress*. Participants were carefully instructed that this data collection was not part of the assessment and that they were expected to answer honestly according to their actual experience, while no such announcement was made for the EES events. To become familiarized with the method, the first point of measurement was before the problem scenario and was not included in the analysis. Six measurement points followed during the problem scenario at minute 4′, 9′, 14′, 19′, 24′, and 29′. Scales were calculated from the six items of each statement. Internal consistencies (Cronbach’s alpha) were C.A. = 0.70 for “interesting,” C.A. = 0.78 for “effort” and C.A. = 0.67 for “progress.”

##### Social desirability

Two measures were applied to investigate whether social desirability played a role in answering the EES items. First, we administered seven items from the scale “impression management” from the “Balanced Inventory of Desirable Responding (BIDR)” (Paulhus, 1994) in a German version by Musch et al. (2002). Paulhus (1994) defined and measured “impression management” as the purposeful deception of looking good to someone. Participants were to rate statements that referred to misconduct that one is usually not willing to admit to such as, for instance, “I sometimes tell lies if I have to” (inverse item) or “I never take things that do not belong to me.” Responses were given on a four-point Likert-scale. The internal consistency (Cronbach’s alpha) was C.A. = 0.71. Second, immediately after the completion of the scenario, the participants completed a short questionnaire. One question aimed at “impression management” during EES responses. Participants had to rate the statement “Concerning the interposed questions, I thought hard about which answer would make me look good” on a five-point scale from 1 = *strongly disagree* to 5 = *strongly agree*.

##### Experience of EES

In the same short questionnaire directly after the problem scenario two additional questions were aimed at assessing the *authenticity* of the EES events (“The interposed questions [phone call, visit to my office etc.] are very realistic”) and the *additional burden* due to the EES events (“I would have arrived at a better solution without these interposed questions [phone call, visit to my office etc.]”).

##### Test motivation

We administered an adapted version of the Effort Thermometer which Kunter et al. (2002) originally developed for and applied in the Programme for International Student Assessment (PISA). The participants were requested to indicate the effort that they had invested in the previous problem scenario on a 10-point scale compared to the maximum effort they would have invested in a test situation of very high personal relevance. The Effort Thermometer was administered directly after the problem scenario.

#### Data Analysis

For correlation analysis, Kendall’s tau-b correlations were calculated because the data were not normally distributed and the sample size was small. The data was analyzed using IBM SPSS 24.

### Results

#### Descriptive Statistics and Social Desirability (RQ1 and RQ2)

**FIGURE A2:**
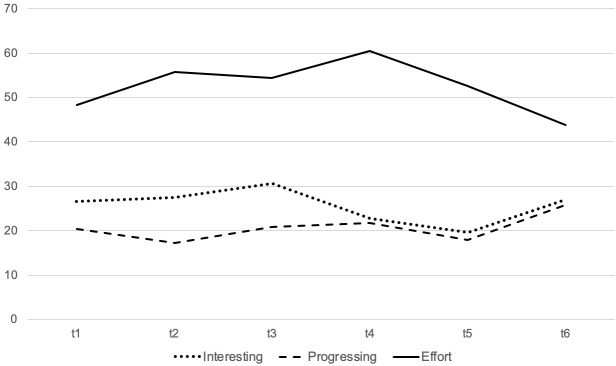
Mean scores of the CSSM items for each measurement point.

On average, the participants experienced the problem scenario as not being very interesting (see EES variable D1 and CSSM scale “interesting”). They invested medium effort according to the CSSM scale “effort” and showed a correspondingly medium test motivation as measured by the Effort Thermometer. With regard to the EES events, the participants did not report that they tried to “look good” when answering the EES items. On average, they experienced the EES events as being quite authentic and hardly as an additional burden (see descriptive statistics in Appendix Table A2). Altogether, the data support H1b and H2b (see Table 2). The average CSSM ratings of “interesting,” “progressing,” and “effort” did not vary very much during the course of the problem scenario. The curve for “effort” resembles an inverted U-shape while ratings of “interesting” and “progressing” increased toward the end of the 30-min problem scenario (see Appendix Figure A2).

#### Relations Between EES Data and CSSM Data (RQ4)

Table 6 shows the correlations of selected EES items and corresponding CSSM items.

**Table 6:** Correlations of selected EES items and corresponding CSSM items in Study 2.

	Correlations with CSSM ‘Progress’						
	MP1	MP2	MP3	MP4	MP5	MP6	Scale
EES C1 (confidence)	0.48	0.17	0.25	0.25	–0.09	0.28	0.21

	**Correlations with CSSM ‘Interesting’**						
	**MP1**	**MP2**	**MP3**	**MP4**	**MP5**	**MP6**	**Scale**

EES D1 (interest)	0.31	0.65	0.32	0.20	0.24	0.44	0.42


*18 < n < 21. Kendal’s tau-b correlations; MP, measurement point.*

Table 6 shows that there are substantial correlations between the Embedded Experience Sampling (EES) and the Continuous State Sampling (CSSM) of situational interest (supporting H4a) while there are smaller correlations between EES data and CSSM data of confidence in one’s competence and subjectively perceived progress, respectively.

#### Relations Between EES Data and Impression Management and Test Motivation (RQ1 and RQ4)

An analysis was made of how far EES data are influenced by social desirability or impression management and how it relates to test motivation. Table 7 shows the results of the respective correlation analysis.

**Table 7:** Correlations between EES items, impression management, and test motivation.

	*(1)*	*(2)*	*(3)*	*(4)*	*(5)*	*(6)*	*(7)*
(1) EES C1 (confidence in one’s competence)	1						
(2) EES C2 (uncertainty tolerance)	0.25	1					
(3) EES D1 (interest)	0.38	0.65	1				
(4) EES D2 (positive emotional states)	0.38	0.08	0.01	1			
(5) Dispositional impression management (BIDR)	–0.06	–0.02	0.05	–0.09	1		
(6) Situational impression management	0.17	0.28	0.22	0.29	0.01	1	
(7) Test motivation (effort thermometer)	0.29	0.57	0.62	0.30	0.17	0.26	1


*17 < n < 21, Kendall’s tau-b correlations.*

As shown in Table 7, there are almost zero correlations between dispositional impression management and the EES variables. Furthermore, there are only small correlations between the EES variables and situational impression management (i.e., having “… thought hard about which answer would make me look good”). There are medium to large correlations between some EES variables and test motivation, which is in line with our theoretical argument. Altogether, the data support H2b and H4b (see Table 2).

## Study 3: Calibration Study of Measuring Non-Cognitive Facets of Competence Via EES

Finally, the computer-based assessment of domain-specific problem-solving competence was implemented in a large-scale study with almost 800 participants in vocational schools in six federal German states. Parts of this final step of the test development are published in Rausch et al. (2016). Hence, parts of the following description are borrowed from Rausch et al. (2016).

### Materials and Methods

#### Participants

A total of 786 VET students participated in the study, of which six were excluded from the analyses due to missing data (due either to lack of willingness or a technical malfunction of the test software). The participating VET students were in the 2nd or 3rd year of a 3-year commercial apprenticeship program, 50.1% were female and the sample showed a typical right skewed age distribution (*M* = 21.3 years; *SD* = 2.69; min = 17; max = 44).

#### Procedure

All data were collected in computer-equipped classrooms in vocational schools. At the beginning of the data collection sessions the researchers introduced the project and the agenda. All participants provided written informed consent. Before and after the problem scenarios, the participants completed several self-report questionnaires including scales on work-related interest and work-related self-concept. In the following, we focus on the internal consistency and internal validity of the assessment of the non-cognitive facets of domain-specific problem-solving competence.

#### Measures

##### Embedded experience sampling (EES)

For the main study, four EES events were defined. The first three EES events were the same that were used in the previous pilot study (see Table 5: short email response after the reception of the task, phone call from the sender of the task, short visit by a colleague). Table 8 only lists the additional fourth EES events, the related competence facets, and the EES items that were used.

**Table 8:** Additional fourth EES events, competence facets, and EES items in Study 3.

EES event (point of time)	Competence facet (see Table 1)	EES items (translated from German and condensed)
EES event 4: short request from the sender of the task after the reception of the solution (after submission or after 30 min)	Situational confidence in one’s solution (C3)	C3: Sender of the task asks how confident the apprentice is about her/his solution and whether the solution has to be checked before its implementation. Answer from 1 = ‘*Unfortunately*, *I did not arrive at a solution at all’* over 2 = ‘*I am afraid you should check everything in detail because I assume I made some mistakes*’ to 5 = *‘I think I found a proper solution that you do not have to check in detail again*.’
	Interest in the progress of/in learning from the problem (D3)	Participants are to check two of the following statements for his email answer. *‘Working on tasks like this, …* D3_1: *… I am always a bit anxious that I might not solve it.’* (*distractor*) D3_2: *… I feel as if I am accepted as a full team member.’* (*distractor*) D3_3: *… I can always learn something interesting.’* D3_4: *… I have the opportunity to demonstrate my skills.’* (*distractor*) D3_5: *… I am afraid to make a fool of myself if I fail.’* (*distractor*) D3_6: *… I wish that afterwards someone would explain to me how I could have done better.’*


##### Generalized self-reports of work-related self-efficacy and work-related interest

We administered a questionnaire on work-related self-efficacy (Abele et al., 2000) which consisted of six statements that had to be rated on a five-point Likert scale ranging from 1 = *disagree* to 5 = *agree* (e.g., “I do not worry about work-related challenges because I can always trust my abilities.”). Cronbach’s alpha was 0.69. An adapted version of a scale to measure dispositional interests in students (Schiefele et al., 1993) was administered to measure dispositional work-related interest. Six statements had to be rated on a four-point Likert scale ranging from 1 = *disagree* to 4 = *agree* (e.g., “I am sure that I have chosen an apprenticeship program which reflects my personal interests”). Cronbach’s alpha was 0.76.

#### Data Analysis

To assess the cognitive facets of competence (see competence model in Table 1), a complex three-step method (similar to Bennett et al., 2003) was applied: (1) Fine-grained results from a highly structured content analysis were condensed into (2) partial credit items on the basis of consensual expert judgments. (3) Finally, these partial credits were subject to psychometric scaling using a multidimensional Rasch model. For further details see Rausch et al. (2016) and Seifried et al. (unpublished).

### Results

#### Requirements of IRT (RQ3)

The variables of non-cognitive facets were calibrated in a six-dimensional partial credit model (Masters, 1982). However, facet D3 (“interest in the progress of/in learning from the problem”), showed insufficient reliability (EAP/PV reliability = 0.30) and therefore was excluded. Thus, the final estimation only included five dimensions and was estimated including background information such as gender, age, vocation, intelligence, competence scores for the cognitive facets, and other relevant variables. All calculations were conducted using the R package TAM (Kiefer et al., 2015). Table 9 shows the EAP/PV reliabilities (on the diagonal) and the latent correlations between the five remaining non-cognitive competence facets (Rausch et al., 2016).

**Table 9:** EAP/PV reliabilities (diagonal) and latent correlations of the non-cognitive facets and generalized self-reports in Study 3.

	(C1)	(C2)	(C3)	(D1)	(D2)
(C1) Situational confidence in one’s competence	0.85				
(C2) Ambiguity/uncertainty tolerance	0.57	0.77			
(C3) Situational confidence in one’s solution	0.72	0.46	0.84		
(D1) Interest in the problem context/content	0.57	0.62	0.38	0.80	
(D2) Maintaining positive and active emotional states	0.51	0.39	0.45	0.45	0.78
Generalized self-report of work-related self-efficacy	0.29	0.25	0.27	0.16	0.21
Generalized self-report of work-related interest	0.24	0.27	0.15	0.27	0.10


*Parts of these results were published in Rausch et al. (2016).*

#### Correlations With Generalized Retrospective Measures (RQ4)

Furthermore, Table 9 shows correlations between non-cognitive facets as measured by EES and the corresponding generalized self-report measures of work-related self-efficacy and work-related interest.

Table 9 shows that the EES data meet the requirements of IRT with the exception of D3 (see above). This supports H3b. There are only small correlations between EES-based scores and scores that are based on generalized self-reports, supporting H4c.

## Discussion

### Summary of Results

Non-cognitive facets of competence are often neglected in competence assessments. In this paper we introduced Embedded Experience Sampling (EES) as an approach to measuring non-cognitive facets of domain-specific problem-solving competence within a computer-based office simulation. The feasibility and validity of EES were investigated throughout three studies by using different measures and analysis approaches. Most of the results support the validity of EES. The results are discussed with regard to the research questions and hypotheses that were outlined previously (see Table 2).

Research question 1 aimed at the test-takers’ perception of the EES events in terms of ecological validity. It was hypothesized that participants in low-stake tests do not experience EES events as an additional and unrealistic burden, a finding supported by group discussions and individual interviews in study 1 and by survey data in study 2. Despite experiencing the scenario as quite difficult, they considered it to be authentic and, on average, did not evaluate EES as an additional burden.

Research question 2 aimed at social desirability as a potential bias in terms of construct validity. In study 1, the participants’ responses in group discussions and individual interviews gave no reasons to assume biases from social desirability. In study 2, the dispositional tendency for impression management was uncorrelated with the EES responses and situational impression management (i.e., having thought about which response to the EES items would make someone look good) showed very small correlations with the EES responses. Altogether, social desirability does not appear as a source of bias in EES responses.

Research question 3 aimed at assessing the consistency of the EES data with assumptions of the Multitrait-Multimethod approach (MTMM) and the requirements of a multidimensional model based on Item Response Theory (IRT) in terms of internal validity. In study 1, low correlations of heterotrait-monomethod combinations and higher correlations of monotrait-heteromethod combinations support the assumption of internal validity, however, the differences are only small. In study 3, the EES data was calibrated in a multidimensional IRT model and showed satisfactory EAP/PV reliabilities for five of the six facets while one facet had to be excluded due to low reliability. Altogether, our analysis supports the assumption of internal validity.

Research question 4 aimed at the correlation of EES data with CSSM data (Continuous State Sampling Method) and test motivation in terms of convergent validity and the correlation between EES data and generalized retrospective self-reports in terms of divergent validity. Substantial correlations between EES data and test motivation support the assumption of convergent validity, while the correlations between EES data and CSSM data were more heterogeneous. Low (almost zero) correlations between EES data and generalized retrospective self-reports in study 1 and study 3 emphasize the significance of the measurement approach.

Altogether, we collected data on the feasibility and validity of EES throughout three field studies on problem-solving competence in the business domain and found very promising results. Embedding self-reports on situational experience into the “storyline” of authentic problem scenarios produces reliable and valid data on non-cognitive facets of problem-solving competence.

### Limitations and Further Research

Both, the methodological approach and the empirical studies have their limitations. First and foremost, we have not tested for external validity, namely by measuring whether emotional states in the test situations are good proxies for emotional states in respective work situations, which constitutes a strong assumption; not only for the non-cognitive facets but also for the cognitive facets of competence. However, it is very difficult to put together an appropriate research design and collect the respective data to investigate these assumptions. Furthermore, data collection in EES is still based on self-reporting. In our studies, we did not find indications of social desirability or of an additional burden due to EES. However, these studies comprised low-stakes testing. In high-stakes testing, responses to EES items are prone to manipulation and EES events might be experienced as more disruptive. The operationalization of the non-cognitive facets of problem-solving competence is arguable. In study 1, the internal consistencies of EES items measuring the same facet were not satisfactory. Many alternative items would have been just as appropriate or maybe more appropriate as indicators of the respective facet. We have not experimented widely with the operationalization of the facets. One significant alteration concerned the facet D3 “interest in the progress of/in learning from the problem.” However, this alteration worsened the model fit and resulted in the exclusion of facet D3 from the IRT model in Study 3, while the correlations within the MTMM analysis in Study 1 had been quite promising. We will vary the item content and the item format in future studies and we encourage other research teams to apply similar approaches in their studies, too.

Limitations of Study 1 and Study 2 were the smaller sample sizes that did not allow for more sophisticated analyses. In Study 2, the CSSM items could have been more similar to the other EES items. Only the items regarding situational interest were very similar. In future studies, more appropriate CSSM items should be applied. Moreover, physiological measures such as heart rate (HR), heart rate variability (HRV), skin conductance or cortisol may be used to further validate the EES data. One such study was conducted by Kärner et al. (2018) who also used the above office simulation and found that CSSM data and physiological data (HR, HRV, and cortisol) showed very similar trends in the course of problem solving. A further data source for validation is the log files from the office simulation. Novak and Johnson (2012) discuss how this non-intrusive data source can be used to measure emotion. Finally, an experimental study in which the participants’ emotional experience is manipulated would allow the sensitivity of EES to be tested.

## Conclusion

Twenty years ago, Weinert (1999) stated that “when assessing competencies, current motivational influences on performance cannot be measured. […] It is feasible only to measure competence-specific motivational attitudes, for example, with reliable and valid questionnaires” (Weinert, 1999, p. 20). In this paper, we introduced Embedded Experience Sampling (EES) as an alternative method to measure non-cognitive facets of competence within the performance assessment instead of relying on decontextualized general self-reports. The idea behind EES is that the repeated measurement of emotional or motivational states during domain-specific tasks allows for an inference to be made regarding non-cognitive traits; similar to Chomsky (1965) distinction between manifest performance and latent competence. This helps to overcome the asymmetry in the content and breadth in the measurement of the cognitive and non-cognitive constructs (Brunswik, 1956).

Drawing on our experience, EES is a feasible and informative approach to measuring non-cognitive facets of competence under the following conditions: (1) The computer-based performance assessment is embedded in an immersive and authentic simulation of a real-life domain. (2) The participants are confronted with comprehensive scenarios that require a sustained performance. (3) The participants are introduced to EES within a tutorial prior to the performance assessment. Drawing on our empirical studies, we found indications of the validity of EES. We would like to encourage other researchers to implement EES or similar approaches into their studies of competence assessment because further research is needed for the subsequent development and validation of the method.

## Ethics Statement

This study was carried out in accordance with the recommendations of the Ethical Board of the University of Bamberg, Germany and the Board of Data Protection of the Federal State Authority of Bavaria (Germany) with written informed consent from all subjects.

## Author Contributions

AR, KK, and JS contributed to method, data collection, and preparation of the manuscript. AR led the project.

## Conflict of Interest Statement

The authors declare that the research was conducted in the absence of any commercial or financial relationships that could be construed as a potential conflict of interest.

## Appendix

**Table A1:** Descriptive statistics of EES items in Study 1.

	Scenario 1		Scenario 2		Scenario 3	
EES variable	*M*	*SD*	*M*	*SD*	*M*	*SD*
**C1** (Situational confidence in one’s competence)	2.16	0.57	2.72	0.56	1.77	0.65
**C3** (Situational confidence in one’s solution)	2.22	0.88	2.97	0.65	1.71	0.96
**D2** (Maintaining positive and active emotional states)	2.48	0.61	3.19	0.46	2.32	0.65
**D3** (Interest in the progress of/in learning from the problem)	1.87	1.06	2.10	1.20	1.83	1.06

*See Table 3 for corresponding EES items.*

**Table A2:** Descriptive statistics of EES items in Study 2.

	Range	Minimum	Maximum	*M*	*SD*
EES variable C1	1–4	1.00	4.00	1.58	0.71
EES variable C2	1–4	1.00	4.00	2.15	0.81
EES variable D1	1–4	1.00	2.00	1.57	0.51
EES variable D2	1–4	1.00	3.25	2.19	0.64
CSSM scale Interesting	1–100	3.83	49.33	24.34	13.62
CSSM scale Effort	1–100	13.17	89.17	52.06	22.34
CSSM scale Progress	1–100	1.17	41.17	19.80	13.61
Impression management (BIDR scale)	1–4	1.14	3.43	2.42	0.56
Impression management (single item)	1–5	1.00	4.00	1.80	0.89
Authenticity of EES events	1–5	1.00	5.00	3.65	1.27
Additional burden of EES events	1–5	1.00	5.00	2.45	1.23
Test motivation (Effort Thermometer)	1–10	3.00	10.00	6.56	2.55

*See Table 5 for corresponding EES items.*
